# Probiotics in the treatment of chronic kidney disease: a systematic review

**DOI:** 10.1590/2175-8239-JBN-3931

**Published:** 2018-06-21

**Authors:** Raquel Aparecida Bandeira Fagundes, Taís Fátima Soder, Kamila Castro Grokoski, Fábia Benetti, Roberta Hack Mendes

**Affiliations:** 1 Universidade Regional Integrada do Alto Uruguai e das Missões, Frederico Westphalen, RS, Brasil.; 2 Universidade Federal do Rio Grande do Sul, Porto Alegre, RS, Brasil.

**Keywords:** Renal Insufficiency, Chonic, Probiotics, Lactobacillus, Insuficiência Renal Crônica, Probióticos, Lactobacillus

## Abstract

Chronic kidney disease (CKD) is a syndrome caused by the progressive reduction of renal function. This study aimed to systematically examine the effects of supplementation with probiotics in the treatment of CKD. Searches were carried out on databases MEDLINE (PubMed), SciELO, Cochrane, and Clinical Trials. Two independent reviewers selected the studies from which data was extracted. The search included papers written in English and Portuguese published in the 2012-2016 period describing randomized clinical trials. Eight of the 82 eligible articles met the inclusion criteria. Sample size ranged from 18 to 101 individuals with CKD. The duration of the included studies varied from four to 24 weeks. Most of the included articles reported positive effects in renal function and decreased levels of urea, blood urea nitrogen, ammonia, plasma p-cresol, p-cresyl sulfate, and indoxyl sulfate.

## RESUMO

A insuficiência renal crônica (IRC) é definida como uma síndrome causada pela redução progressiva da função renal. O objetivo deste trabalho foi revisar sistematicamente o efeito da suplementação probiótica no tratamento da IRC. Foi realizada uma busca nas bases de dados MEDLINE (PubMed), SciELO, Cochrane e Clinical Trials. Dois revisores independentes realizaram a seleção dos estudos e a extração de dados. A pesquisa incluiu estudos entre 2012-2016, do tipo estudo clínico randomizado, em inglês e em português. Dos 82 artigos elegíveis, 8 artigos preencheram os critérios de inclusão. O número amostral variou de 18 a 101 pacientes com IRC, com duração de 4 a 24 semanas de estudo. A maioria dos estudos relatados mostraram efeitos benéficos na redução das concentrações de ureia, nitrogênio ureico, amônia, p-cresol plasmático, sulfato de p-cresil e sulfato de indoxil, ou seja, os probióticos parecem estar relacionados à melhora da função de renal.

## INTRODUCTION

Chronic kidney disease (CKD) may be defined as a syndrome caused by the progressive reduction of renal function.^1^ It is characterized by progressive deterioration of organic biochemical and physiological function secondary to the accumulation of catabolites, disturbed fluid-electrolyte and acid-base balance, metabolic acidosis, hypovolemia, hyperkalemia, hyperphosphatemia, anemia, hormonal disorders, hyperparathyroidism, infertility, and growth failure, to name a few.^2^^,^ 3 ^,^ 4

More than 20 million people have kidney disorders in the United States, and more than 600,000 suffer from kidney failure.^5^ In Brazil an estimated 10 million people have some degree of renal disorder, while the global incidence of kidney disease grows at a mean rate of 10% a year.^6^ The number of individuals affected by kidney disease has increased significantly, partly on account of population aging and partly due to the growth in the number of people with hypertension and *diabetes mellitus*, two morbidities strongly correlated with the development of renal involvement.^7^^,^ 8

Patients with CKD and end-stage renal disease (ESRD) present quantitative and qualitative alterations in the gut microbiota such as increased concentration of urea and ammonia in the bowel, compromised integrity of the intestinal barrier, and increased levels of inflammation.^9^ Considering the bacteria living in the lower gastrointestinal (GI) tract, an estimated 100 trillion microorganisms live in the human bowel,^10^ a number ten times greater than the number of cells in a living organism. The members of this microbiome play a key role in immune response development and function.^11^

Probiotics include a vast array of products with living microorganisms^12^ whose purpose is to improve intestinal microbial balance and produce beneficial effects on one's health.^13^ To do so, they must be administered in proper dosages.^14^ In order for probiotics to produce actual benefits to one's organism, they must be viable when used, i.e., the microorganisms in them have to survive contact with gastric juice and bile, to then fixate in the intestinal lining to compete against pathogenic microorganisms and satisfactorily modulate inflammation and immunity.^15^

Patients with CKD with increased plasma levels of uremic solutes in need of kidney transplantation or chronic dialysis have in probiotics an alternative therapy to treat ESRD and attenuate uremia.^11^

The effects arising from the use of probiotics by patients with CKD are unclear, and few studies have been carried out in this area. This systematic review was based on the following guiding question: "Are probiotics beneficial when used as an adjuvant element in the treatment of CKD?" This systematic review aims to examine the effects of probiotics used in the treatment of patients with CKD.

## MATERIALS AND METHODS

### SEARCH FOR LITERATURE AND SELECTION OF STUDIES

The search for literature was carried out from December of 2016 to May of 2017 on the following databases: Scientific Electronic Library Online (SciELO), MEDLINE (accessed via PubMed), Clinical Trials, and Cochrane Library. A search for more recent papers published since May of 2017 was made on June of 2017. An additional search was made on Google Scholar to make sure no relevant paper was missed. The searches were carried out using the following terms: (Chronic renal disease) OR (Chronic kidney disease) OR (Chronic renal failure) OR (Chronic kidney failure) AND (Lactobacillus) OR (Lactobacilli) OR (Probiotic) OR (Probiotics). All terms were used in the searches without using quotation marks (").

### INCLUSION AND EXCLUSION CRITERIA

This systematic review included studies published between 2012 and 2016 in the English and Portuguese languages, designed as randomized clinical trials (RCT) enrolling humans to examine the effect of probiotic supplements, independent clinical trials phases I, II, III, IV enrolling patients with CKD, aged 18 years or older of both sexes, on hemodialysis or not. Review papers, systematic, narrative and integrative reviews, editorials, book chapters and abstracts, experimental trials with animals, and abstracts published in meeting proceedings were excluded.

### REFERENCE PROTOCOL

**Figure 1:**
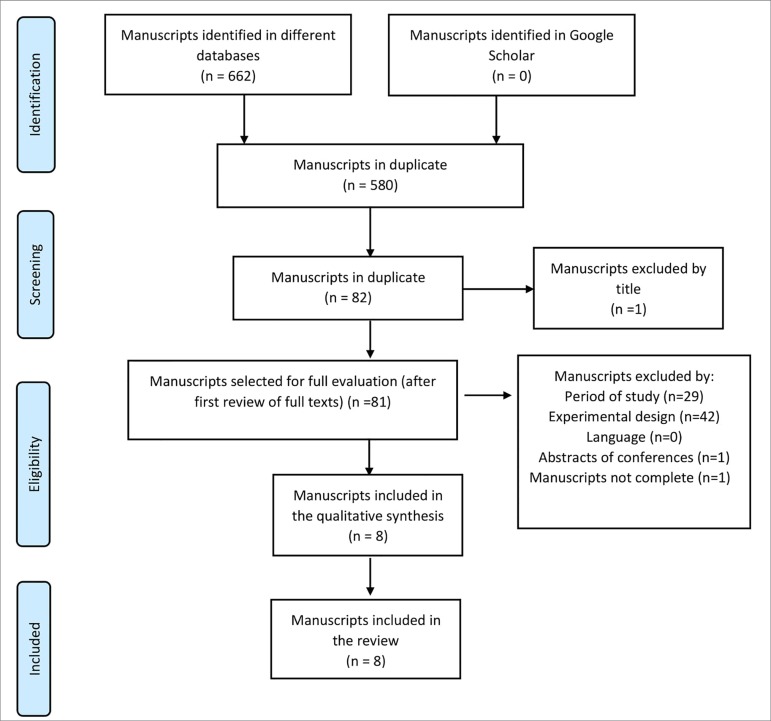
Flowchart describing the identification and selection of articles (PRISMA).

This study was based on the Preferred Reporting Items for Systematic Reviews and Meta-Analyses (PRISMA - Figure 1).^16^ A protocol for this review as registered with PROSPERO (CRD42017064068). As described in Carvalho *et al*.,^17^ "risk of bias" was assessed for each RCT included in the systematic review through a protocol built in accordance with the recommendations of the Cochrane Collaboration.^18^

## DATA EXTRACTION

Two independent reviewers (R.A.B.F. and T.F.S.) searched the databases for papers and selection was made based solely on the titles of the articles. The abstracts of the selected studies were read and assessed based on the eligibility criteria. The disagreements between the two reviewers were discussed until consensus was reached.

## RESULTS

### SEARCH FOR LITERATURE AND SELECTION OF STUDIES

Database searches yielded 662 articles, 580 of which were duplicates. After the analysis of titles and abstracts, 75 records were excluded for not meeting the inclusion criteria and eight were selected and read in their entirety (Figure 1). The eight papers selected were on PubMed. All the studies included in this review were RCTs written in English. The selected studies were performed in Mexico^19^^,^ 20, Italy^21^, the United States^22^, Malaysia^23^, China^24^, Iran^25^ and Australia^26^.

### SAMPLE SIZE, STUDY DURATION, INTERVENTION CHARACTERISTICS

Sample size ranged between 18 and 101 patients with CKD, and study duration varied from four to 24 weeks. Probiotic agent dosages ranged from 2.0 x 10^12^ to 16 x 10^9^ CFU and 15g; dosages did not follow a specific standard. The form of presentation of the probiotic agents varied significantly; some were administered in bags/envelopes to be dissolved in water, while others had to be added to yogurt and a few were given in capsules. In all studies, groups of patients were prescribed probiotics in different presentations - capsules, bags, etc. - and times in relation to meals -right after or while eating.

### ASSESSMENT OF STUDY RISK OF BIAS

Table 1 describes the methodological quality of the included studies. The only criterion established by the Cochrane Collaboration to assess methodological quality referred to other sources of bias, an area in which all studies presented low levels of risk. However, all studies were designed to answer clear focal questions. Five studies offered detailed descriptions of the method used to generate random sequences, while three described in detail the method used to hide patient allocation. The methods used to define the blinding of patients or researchers were reported in seven studies. Seven studies were described as blinded experiments, but failed to describe all the measures taken to enforce blinding. Results were completely assessed in seven of the eight studies.

**Table 1:** Assessment of risk of bias in randomized clinical trials (Cochrane Collaboration)

Study	Randomization	Allocation	Blinding of participants or researchers	Blinding for outcomes	Incomplete outcomes (losses)	Selective outcome report	Other sources of bias
Alatriste *et al.*, 2014	↓	↑	↑	↑	↑	↓	↓
Mora *et al*., 2014	?	?	↓	↓	↓	↓	↓
Guida *et al*., 2014	↓	↓	↓	↓	↓	↓	↓
Natarajan *et al*., 2014	?	?	↓	↓	↓	↑	↓
Firouzi *et al*., 2015	↓	↑	↓	↓	↑	↓	↓
Wang *et al*., 2015	↓	↓	↓	↓	↓	↓	↓
Dehghani *et al*., 2015	?	?	↓	↓	↑	↓	↓
Rossi *et al*., 2016	↓	↓	↓	↓	↓	↓	↓

↑: High risk; ↓: Low risk; ?: Unknown risk.

### TYPES AND DURATION OF THE INTERVENTIONS

Only one study tested the effects of yogurt with added probiotics;^19^ another study tested a protocol with one daily symbiotic gel capsule.^20^ Probiotics were also presented in envelopes dissolved in water and ingested three times a day, not on meal times.^21^ Natarajan *et al*.^22^ prescribed two capsules three times a day ingested during meals. Bags with probiotics dissolved in water and ingested promptly were also prescribed twice a day (in the morning and in the evening) accompanied or not by a meal.^23^ Wang *et al*.^24^ prescribed one capsule a day before going to bed. Dehghani *et al*.^25^ prescribed two capsules a day after meals, while Rossi *et al*.^26^ offered 15g of probiotics daily in a symbiotic formulation.

### MOST PREDOMINANT MICROORGANISMS USED IN PROBIOTIC AGENTS

Most of the microorganisms used in the studies belonged to the *Lactobacillus* (100% = 8 studies) and *Bifidobacterium* genera (87.5% = 7 studies). Table 2 shows the data extracted from the selected studies.

**Table 2:** Characteristics of the studies included in the review

Study	N	Treatment duration	Probiotic strain	Dosages	Beneficial effects	Side effects	Nutritional counseling	Outcome
Alatriste *et al*. (2014)	30	8 weeks	*Lactobacillus casei Shirota (LcS)*	Group A was given a fermented dairy drink in an 80-mL bottle with 8 x 109 CFU of LcS; Group B was given two 80-mL bottles de 80 mL of a fermented dairy drink with 16 x 109 CFU of LcS.	Yes. The patients presented decreased levels of ammonia (a precursor of urea, with which bacteria are involved).'	None	Yes	Decrease greater than 10% in serum urea levels after dietary intervention with LcS for patients with CKD stages 3 and 4.
Mora *et al*. (2014)	18	8 weeks	*Lactobacillus acidophilus and Bifidobacterium bifidum*, prebiotic inulin fiber, omega-3 fatty acids, and vitamins (B vitamins, folic acid, ascorbic acid, and vitamin E).	Nutrihealth: 1 capsule/day – 2.0 x 10^12^ CFU	Yes. Increased counts of bifidobacteria.	None	Yes	Symbiotic gel used for two months may be potentially in the therapy of patients with kidney disease and may increase the population of bifidobacteria.
Guida *et al.* (2014)	30	4 weeks	*Lactobacillus plantarum, Lactobacillus casei subsp. Rhamnosus, Lactobacillus gasseri, Bifidobacterium infantis, Bifidobacterium longum, Lactobacillus acidophilus, Lactobacillus salivarius, Lactobacillus sporogenes, Streptococcus thermophilus, previotic inulin (VB Beneo Synergy 1),* and *resistant tapioca starch.*	Probinul neutro^®^ (5g powder bags dissolved in water, three times a day, away from meal times).	Yes. The symbiotic agent significantly decreased plasma p-cresol levels of non-dialysis patients with CKD.	None	None	Symbiotic agent may significantly decrease total plasma p-cresol levels in patients with CKD stages 3 and 4.
Natarajan *et al.* (2014)	22	24 weeks	*S. thermophilus KB 19, L. acidophilus KB 27, B. longum KB 31.*	Two capsules three times a day with meals. Each capsule contained a probiotic formulation with 30 billion CFU.	None.	Yes	None	Efficacy could not be confirmed primarily due to small sample size and low statistical power - additional studies are needed.
Firouzi *et al*. (2015)	10 1	12 weeks	*Lactobacillus acidophilus, Lactobacillus casei, Lactobacillus lactis, Bifidobacterium bifidum, Bifidobacterium longum* and *Bifidobacterium infantis.*	Daily dosage of 6 × 1010 CFU (Hexbio B-Crobes). Probiotic agent in bags were poured into a glass with approximately 250 mL of water; twice a day (morning and evening) accompanied or not by a meal.	Yes Urea levels improved significantly after supplementation with probiotics.	Yes	Yes	This study showed that probiotics may improve urea levels, particularly of OW/OB individuals and subjects with high urea levels. However, other renal parameters and liver function were not altered by the administration of probiotics.
Wang *et al.* (2015)	39	24 weeks	*B. bifidum A218, B. catenulatum A302, B. longum A101, and L. plantarum A87.*	Daily dosage: 1 capsule 4 × 109 CFU/day before going to bed.	Yes. Significant decreases were seen in the levels of TNF-α, IL-5, IL-6, and endotoxins, along with increased levels of IL-10; residual renal function of patients on PD was preserved after six months of treatment with oral probiotics.	None	None	Probiotics may significantly reduce serum levels of endotoxins, proinflammatory cytokines (TNF-α and IL-6), IL-5, and increase serum levels of proinflammatory cytokines (IL-10) and preserve residual renal function of individuals on PD.
Dehghani *et al.* (2016)	66	6 weeks	*Lactobacillus casei, Lactobacillus cidophilus, Lactobacillus bulgarigus, Lactobacillus rhamnosus, Bifido,bacterium breve, Bifidobacterium longum, Streptococcus thermophilus* and fructooligosaccharide prebiotic agent.	Familact 500mg - two capsules a day after meals.	Yes. Blood urea nitrogen levels of patients with CKD were decreased.	None	None	Treatment with symbiotic probiotics for six weeks led to significant decreases in mean blood urea levels of patients with CKD stages 3 and 4 compared to controls; effects were not seen in other renal function indicators.
Rossi *et al*. (2016)	31	18 weeks	Nine different strains from the *Lactobacillus, Bifidobacterium* and *Streptococcus genera*. High molecular weight inulin, fructooligosaccharides, and galacto-oligosaccharides, and probiotic component.	In the first three weeks, participants ingested 7.5g of prebiotic powder and one capsule with probiotics containing 45 billion CFU in the morning with a meal. In the last three weeks, participants took an additional dose (7.5 g of powder and one capsule) with a meal in the evening, yielding a daily dosage of 15g.	Yes. Symbiotic therapy significantly decreased serum levels of PCS and, to a lesser extent, IS levels in patients with moderate to severe CKD.	None	Yes	Symbiotic therapy led to statistically significant and potentially clinically relevant decreases in serum levels of IS and PCS.

CFU: colony forming unit; LcS: Lactobacillus casei Shirota; PCS: p-cresyl sulfate; IS: indoxyl sulfate; OW: overweight; OB: obese; PD: peritoneal dialysis. CKD: chronic kidney disease.

### RENAL FUNCTION MARKERS

Renal function biomarkers such as serum creatinine, urea, ammonia, and uric acid were analyzed in six studies.^19^^,^ 22 ^-^ 26 Positive effects were reported for some renal function markers based on decreases in blood urea nitrogen levels.^19^^,^ 25 Other markers did not decrease significantly (creatinine, creatinine clearance, uric acid, glomerular filtration rate, sodium, potassium) before or after the intervention.^22^^,^ 23 ^,^ 24 ^,^ 25 ^,^ 26

### INFLAMMATORY MARKERS

Inflammatory biomarkers such as C-reactive protein (CRP) and interleukins were assessed in three studies.^22^^,^ 24 ^,^ 26 When compared to controls, decreases were reported in endotoxins and proinflammatory cytokines TNF-α, IL-5, IL-6, and increases in serum IL-10 levels.^24^ Narajan^22^ did not report changes in inflammatory marker concentrations as a result of probiotic administration. Rossi *et al*.^27^ reported that probiotic therapy produced positive effects on the serum levels of free and total p-cresol sulfate and indoxyl sulfate.

## DISCUSSION

This systematic review revealed the main microorganisms targeted in the treatment of CKD. The list includes bacteria belonging to the *Lactobacillus* and *Bifidobacterium* genera, with reported beneficial effects such as decreasing urea, blood urea nitrogen, and ammonia levels and plasma concentrations of p-cresol and indoxyl sulfate. Another benefit described for these probiotic strains is that they reportedly help increase bifidobacteria populations, a genus known to play a key role in the function of the intestinal mucosal barrier^26^ and to help decrease cytokine and endotoxin concentrations and increase serum levels of IL-10. Other strains, such as the ones belonging to genera *S. thermophilus KB 19, L. acidophilus KB 27, B. longum KB 31,* did not produce beneficial effects to human health.

Alatriste *et al*.^19^ observed that higher dosages of probiotic agents with LcS yielded better outcomes. Probiotic dosages in the range of 16 x 10^9^ CFU administered for eight weeks in combination with diet and protein intake led to decreases in blood urea levels. The benefits of offering probiotics to patients with CKD included a protective effect stemmed from decreased levels of inflammatory markers. Natarajan *et al*.^22^ reported that the administration of Renadyl to patients with CKD at a dosage of 180 billion CFU/day was safe and well tolerated. Non-significant trends were observed in white blood cell count, CRP, total indoxyl glucuronide, uremic toxins, markers of oxidative stress, and quality of life measures.

Investigations on the effects of a multi-strain microbial cell preparation on the renal profiles and liver function of individuals with diabetes type 2 for 12 weeks revealed that probiotics might potentially improve urea levels, particularly in overweight and obese individuals with elevated urea levels. Blood sodium and potassium levels and liver function tests remained unaltered after supplementation with probiotics, while creatinine levels increased in case and control groups, a finding possibly related to the administration of angiotensin-converting-enzyme inhibitors. Contrastingly, urea levels decreased in the group offered probiotics. A few adverse effects were reported, including expected minor gastric disorders, sexual impotence, and a carbuncle in one individual.^23^

Supplementation with probiotics appears to be associated with significant decreases in serum levels of proinflammatory cytokines TNF-α, IL-5, and IL-6 and endotoxins in patients on PD, and increased levels of anti-inflammatory cytokine IL-10, in addition to preservation of residual renal function after six months of oral supplementation; urea, creatinine, and uric acid levels remained unaltered after probiotic administration.^24^

The administration of probiotics (Familact 500, twice a day after meals for six weeks) reportedly decreased blood urea levels in patients with CKD stages 3 and 4, although no effects on other indicators of renal function were observed.^25^

Symbiotic supplementation also yielded positive effects to the gut microbiota of patients with CKD. A prescription of one symbiotic capsule for eight weeks increased *Bifidobacterium* and preserved *Lactobacillus* populations;^20^ supplementation for four weeks with probiotics given three times a day away from meal times led to considerable decreases in the plasma p-cresol levels of patients with CKD stages 3 and 4,^21^ while supplementation with 15g a day of symbiotic probiotics resulted in effective decreases in serum p-cresol levels and lesser decreases in indoxyl sulfate levels of patients with moderate to severe CKD treated for 18 weeks.^27^

Choosing the right strain might help maintain the balance between the different populations of microorganisms and thus decrease the potential for overgrowth and pathogenicity.^28^ When the populations of pathogenic bacteria increase, patients with renal impairment develop dysbiosis, which may contribute to increase the levels of uremic toxins often associated with progression of CKD.^29^ Gut colonization is highly affected by eating habits, host phenotype, mental health, and host living habits.^30^

With a growing number of lifestyle-related diseases, healthy eating habits and physical exercise have become more important than drug therapy.^8^ Diet is one of the better known lifestyle factors and an important regulator of the intestinal microbiota.^31^^,^ 32 Dietary intervention is an adjuvant strategy used to restore microbial balance and suppress circulating levels of p-cresol and indoxyl sulfate,^33^ as described in the study published by Rossi *et al*.^27^

However, studies indicated that diet has a strong impact on colonic bacterial metabolism and on the progression of CKD. When associated with dietary fiber in the treatment of CKD, benefits are expected in relation to the integrity of the gastrointestinal wall, along with decreases in the systemic concentrations of hazardous uremic toxins.^34^ Isocaloric (30 kcal/kg of ideal body weight) e isoprotein (0.8 g/kg of ideal body weight) diets benefit patients with renal impairment on account of the impacts they may produce primarily on serum urea levels, a parameter significantly affected by protein intake.^19^

Interventions with clearly presented dietary factors shed light on the possible causal relationships between diet and disease mediated by the intestinal microbiota. The administration of probiotics and prebiotics may be seen as a preventive or therapeutic measure, since it promotes the establishment of a healthier, better functioning intestinal microbiota.^30^

The limitations of this review include the short follow-up period featured in some studies and the usually small size of the enrolled populations. Another limitation was the methodological heterogeneity of the studies, which precluded the production of a meta-analysis. Therefore, this review was limited to a descriptive presentation of the data.

## FINAL CONSIDERATIONS

This systematic review stressed the relevance of probiotics in decreasing urea, blood urea nitrogen, ammonia, plasma p-cresol, IS, PCS levels of individuals with CKD. The main strains used in the treatment of patients with CKD belonged to the *Lactobacillus* and *Bifidobacterium* genera, with dosages ranging from 2.0 x 10^12^ to 16 x 10^9^ CFU and 15g; dosages did not follow a specific standard. Some of the probiotic agents were administered in bags/envelopes dissolved in water, while other agents were given in capsules and one was added to yogurt. Each of the formulations had specific ingestion instructions devised to preserve the properties of the probiotic agents. The limited number of articles on the topic precluded the generalization of the obtained results. Therefore, additional long-term studies are needed to further elucidate the possible role of probiotics in the treatment of individuals with CKD.
